# Pancreatitis, Panniculitis, and Polyarthritis (PPP) Syndrome in the Setting of Severe Acute Pancreatitis

**DOI:** 10.7759/cureus.35768

**Published:** 2023-03-04

**Authors:** Houssem Ammar, Fathia Harrabi, Mohamed Amine Said, Rahul Gupta, Ali Ben Ali

**Affiliations:** 1 Gastrointestinal Surgery, Sahloul Hospital, University of Sousse, Sousse, TUN; 2 Gastrointestinal Surgery, Synergy Institute of Medical Sciences, Dehradun, IND

**Keywords:** general surgery, fat necrosis, arthritis, panniculitis, pancreatitis

## Abstract

The coexistence of lobular panniculitis, polyarthritis, and intraosseous fat necrosis in patients with acute pancreatitis is commonly referred to as pancreatitis, panniculitis, and polyarthritis (PPP) syndrome. It is a rare condition associated with serious complications and high mortality. A 70-year-old female was admitted with severe acute necrotizing pancreatitis due to gallstone disease. Laboratory findings indicated an intense systemic inflammatory response syndrome (SIRS). The patient rapidly progressed toward persistent organ failure. During her hospital stay, she also developed panniculitis and polyarthritis, related to severe acute pancreatitis. Eventually, the patient expired despite medical therapy.

## Introduction

The coexistence of pancreatitis, panniculitis, and polyarthritis (PPP) is a rare condition encountered in patients with a pancreatic disease, known as the PPP syndrome [1-3]. In a literature review, we found that panniculitis and/or polyarthritis is often the first symptom of underlying pancreatic disease. Moreover, few cases have been reported to be associated with severe acute pancreatitis.

Here, we present a case of PPP syndrome with a fatal outcome. The patient presented acute pancreatitis and developed an intense systemic inflammatory response syndrome (SIRS), followed by persistent multi-organ failure. Panniculitis and polyarthritis are initially misdiagnosed but later diagnosed two weeks after admission. Cutaneous lesions were prevalent on the trunk and upper and lower limbs, and polyarthritis was predominant on distal joints. The clinical course of our patient illustrates the severity of PPP syndrome and emphasizes the importance of a multidisciplinary approach to treating this syndrome.

## Case presentation

A 70-year-old female presented to the emergency department with a sudden onset of severe epigastric abdominal pain radiating to the back for two days. She also complained of fever and bilious vomiting for 24 hours prior to her admission. On physical examination, her vital signs were as follows: blood pressure of 94/50 mmHg, pulse rate of 112 beats per minute, respiratory rate of 24 cycles per minute, temperature of 39°C, and 92% oxygen saturation on room air. She was anicteric with marked tenderness in the epigastric region.

Biological tests revealed serum lipase of 3,546 U/L, C-reactive protein levels of 354 mg/L, white blood cells of 16,390/mL, and blood urea nitrogen (BUN) level of 22 mmol/L. Arterial blood gas analysis revealed metabolic acidosis with a pH of 7.26 and bicarbonate (HCO3) of 19 mmol/L. The liver function test showed an aspartate aminotransferase level of 358 UI/L and an aspartate transaminase level of 495 UI/L, with a normal blood bilirubin level. The SIRS score was 4. The bedside score was 4 (blood urea nitrogen level of 22 mmol/L, Glasgow Coma Scale score of 15/15, presence of pleural fluid, SIRS score of more than 2, age of 70 years, and >60 years). The provisional diagnosis of severe acute pancreatitis was made, and the patient was started on intravenous fluid resuscitation, nasogastric decompression, and prophylactic antibiotics. Abdominal ultrasound revealed gallstones and a bulky pancreas.

Contrast-enhanced computed tomography of the abdomen showed an edematous pancreas with peripancreatic fat stranding and non-enhancement of more than 50% of the pancreatic parenchyma.

**Figure 1:**
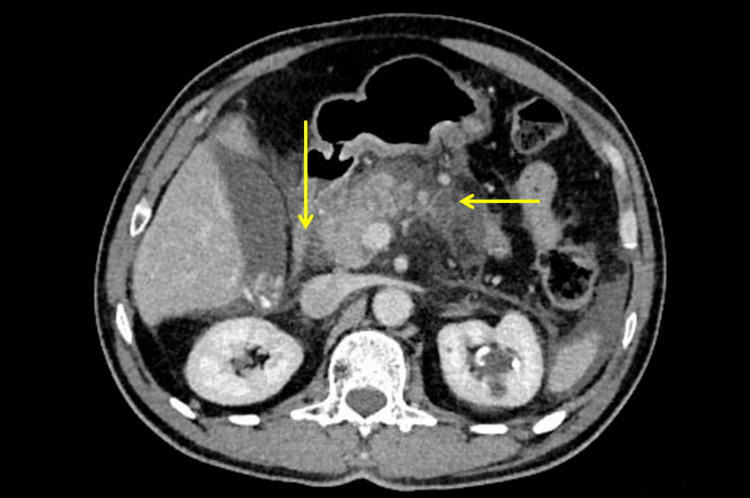
Abdominal contrast-enhanced computed tomography showing pancreatic necrosis and peripancreatic fat stranding (yellow arrows).

Several collections were evident in the intrapancreatic and peripancreatic regions with the largest collection measuring 50 mm in diameter. The superior mesenteric vein was partially thrombosed, and there were ascites and bilateral pleural effusion (left more than right). The Baltazar grade was E, and the computed tomography severity index score was 10 (Figure 1).

**Figure 2:**
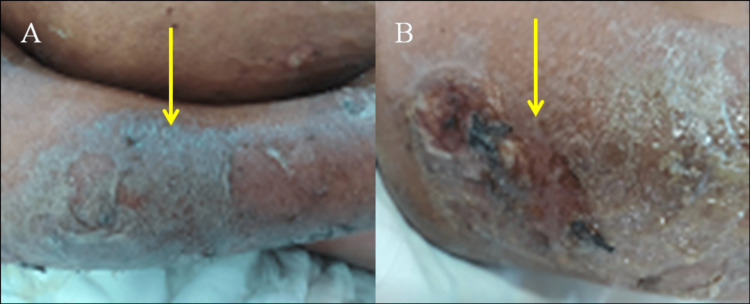
Erythematous, ulcerated cutaneous lesions exuding an oily, brown, and viscous substance due to liquefactive necrosis on the upper limb (yellow arrows) suggestive of panniculitis: arm (A) and elbow joint (B).

During the hospital stay, the patient developed respiratory distress requiring intensive care with mechanical intubation, inotropic support, intravenous fluids, antimicrobials, and parenteral or enteral nutrition. On the 15th day of her hospital stay, we noticed erythematous, ulcerated skin lesions exuding oily, brown, and viscous substances due to liquefactive necrosis. The skin lesions were located on the trunk and upper and lower limbs, and in front of the joints, especially distal joints (Figure 2).

**Figure 3:**
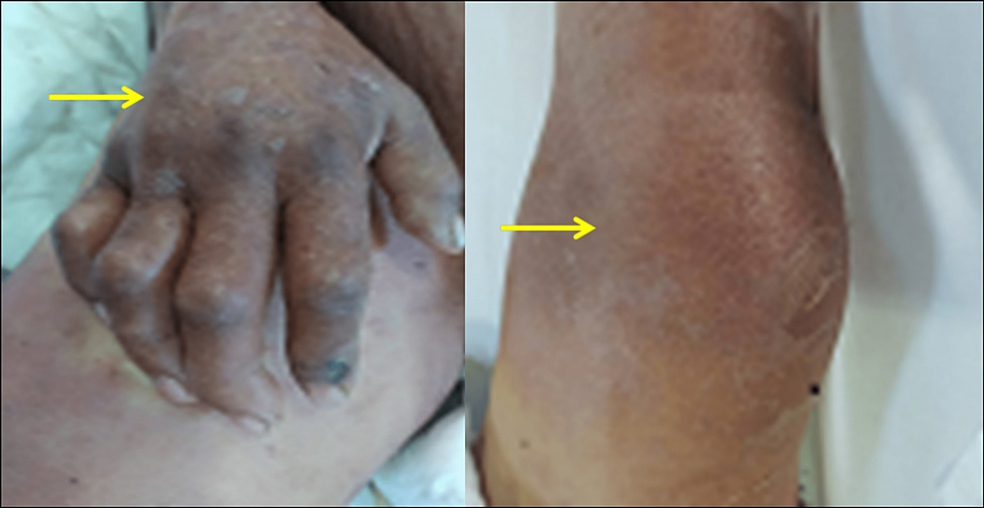
Swollen, edematous, and deformed joints of the hand (A) and knee (B) suggestive of polyarthritis (yellow arrows).

We also noticed deformed and swollen joints suggestive of polyarthritis (Figure 3).

## Discussion

The triad of symptoms, pancreatitis, panniculitis, and polyarthritis (PPP), constitutes a syndrome of extra-pancreatic manifestations in the setting of pancreatic pathology. Pancreatic disease includes acute and chronic pancreatitis, pancreatic carcinoma, and other pancreatic abnormalities [4]. This syndrome is extremely rare with an incidence of 0.3%-3% across a range of different pancreatic diseases [5,6]. The mortality rate of PPP syndrome has been reported to be up to 24%, which is primarily due to the underlying pancreatic disease [7].

Pancreatic panniculitis (PP) is a rare variant of panniculitis characterized by subcutaneous fat necrosis with painful, tender, erythematous to a violaceous nodule that may progress to ulceration and discharge of an oily, brown, viscous material. The lesion may involve the trunk, upper and lower limbs in the front of the joint, and especially the distal joints [6]. In the index case, the skin lesions (panniculitis) were spread in the trunk, arms, hand, feet, and peri-articular regions. Arthritis is also a rare extra-pancreatic complication of pancreatitis. It can affect single or multiple joints. Polyarthritis is often symmetric and affects the distal joints as seen in the present case. The persistent high lipase level was consistent with active pancreatic inflammation and the release of pancreatic enzymes into the systemic circulation. This may cause systemic fat necrosis in the subcutaneous (panniculitis) fat tissue of the skin, bones, and joints (polyarthritis) [4,5,8].

Ideally, for a better and more robust diagnosis, a biopsy and histological analysis are crucial, along with other serological diagnostic tests to rule out other diseases affecting the joints and skin. Histologically, the lesions are characterized by lobular panniculitis without vasculitis. Adipocytes that have undergone coagulative necrosis are called “ghost adipocytes” because they have lost their nuclei [5,9]. However, these tests could not be done due to cost constraints and the critical illness of the patient.

The key to the management of PPP syndrome is the treatment of the underlying pancreatic disease as soon as possible, and this appears to rely on the early diagnosis of this condition [5,7,9]. Nonsteroidal anti-inflammatory drugs and steroids have been seen to relieve the symptoms with partial response. The multi-organ involvement in PPP syndrome requires a close multidisciplinary collaboration [2,7]. The prognosis of this condition depends upon the duration of the illness and worsens with a delay in the diagnosis and the specific treatment of the underlying pancreatic disorder.

## Conclusions

PPP is a rare condition associated with serious complications and high mortality. The tragic clinical course of this patient highlights the importance of the early recognition of the clinical manifestation of pancreatic disease. In fact, even in the absence of histological analysis, the diagnosis could only rely on clinical and blood tests.
